# Evaluation de la fonction hépatique au cours du paludisme grave chez les enfants de moins de cinq ans à Kinshasa en République Démocratique du Congo

**DOI:** 10.11604/pamj.2014.19.266.4673

**Published:** 2014-11-11

**Authors:** Arsène Tshikongo Kabamba, Olivier Mukuku, Laurent Kwete Shamashanga, Daniel Badibanga Kamunga, Alex Impele Bokanya, Zet Kalala Lukumwena, Albert Otshudi Longanga

**Affiliations:** 1 Faculté des Sciences Pharmaceutiques, Université de Lubumbashi, République Démocratique du Congo; 2 Faculté de Médecine, Université de Lubumbashi, République Démocratique du Congo; 3 Faculté de Médecine Vétérinaire, Université de Lubumbashi, République Démocratique du Congo; 4 Université Libre de Bruxelles (ULB), Belgique

**Keywords:** Paludisme grave, fonction hépatique, enfants, severe malaria, liver function, children

## Abstract

**Introduction:**

Le paludisme est toujours compté parmi les problèmes de santé publique prioritaires en République Démocratique du Congo suite au nombre de malades et de décès qu'il provoque. Cette étude évalue l'atteinte de la fonction hépatique au cours du paludisme grave chez les enfants de moins de 5 ans.

**Méthodes:**

Il s'agit d'une étude cas-témoins menée de janvier à juin 2013 à Kinshasa (République Démocratique du Congo) où le dosage des bilirubines totale, directe et indirecte et la mesure de l'activité enzymatique de la Glutamate Pyruvate Transaminase (GPT), de la Glutamate Oxaloacétate Transaminase (GOT) et du taux d'hémoglobine ont été faits chez 46 enfants âgés de moins de 5 ans atteints de paludisme grave (groupe I) et chez 46 autres considérés sains avec une goutte négative (groupe II). Les résultats obtenus ont été comparés dans les deux groupes et le seuil de signification a été fixé à p <0,05.

**Résultats:**

Les analyses statistiques relèvent que les valeurs sont considérablement élevées en ce qui concerne les deux transaminases (GOT et GPT), la bilirubine directe, la bilirubine indirecte et la bilirubine totale chez les enfants atteints du paludisme grave. Ces analyses montrent une différence significative en défaveur de ces derniers (p < 0,001).

**Conclusion:**

En effet, cette augmentation des taux plasmatiques des paramètres biologiques analysés observée chez les enfants gravement impaludés traduit ainsi une altération de la fonction hépatique au cours d'un paludisme grave chez l'enfant de moins de cinq ans.

## Introduction

Le paludisme est une maladie qui peut être mortelle. Il est dû à des parasites transmis à l'homme par des piqûres de moustiques infectés. Il a été estimé au delà de 500 million de peuple souffrant du paludisme chaque année, 1 à 2 million de morts dont 90% sont des enfants en Afrique subsaharien [1]. Selon les dernières estimations de décembre 2013, on a enregistré, en 2012, 207 millions de cas de paludisme (avec une marge d'incertitude comprise en 135 millions et 287 millions) qui ont causé 627000 décès (avec une marge d'incertitude comprise entre 473000 et 789000), soit une diminution de la normalité de 45% au niveau mondial par rapport à 2000 et de 49% dans la région africaine de l'OMS. La plupart de décès surviennent chez des enfants vivant en Afrique où chaque minute un enfant meurt du paludisme. En Afrique le taux de mortalité des enfants a diminué de 54% par rapport l'an 2000 [2].

Cependant, malgré la régression du paludisme au cours des dernières années dans toutes les régions du monde; les chiffres de morbidité et de mortalité restent toujours alarmants, et rend le paludisme la maladie parasitaire tropicale la plus importante. Elle concerne 36% de la population mondiale dans plus de 90 pays ou territoires. Plus de 90% des cas et des décès concernent l'Afrique sub-saharienne, en particulier chez les enfants de moins de 5 ans [1, 3, 4]. Les femmes enceintes et surtout les enfants dont l’âge varie de 4 mois à 4 ans sont les plus touchés par cette parasitose sous sa forme grave avec des complications multi viscérales innombrables dont les mécanismes physiopathologiques sont peu ou mal connus causant ainsi chez les enfants plus de 1 millions des décès par an [1, 5, 6]. Le paludisme sous sa forme grave s'accompagne de graves conséquences sur plusieurs organes (cerveau, rein, foie,…) conduisant à des défaillances fonctionnelles graves de ces organes [7–9]. Cette étude vise à évaluer l'atteinte de la fonction hépatique au cours du paludisme grave chez les enfants de moins de 5 ans à Kinshasa (RDCongo) par le dosage des bilirubines totale, directe et indirecte et par la mesure de l'activité enzymatique de la Glutamate Pyruvate Transaminase (GPT), de la Glutamate Oxaloacétate Transaminase (GOT).

## Méthodes

Il s'est agi d'une étude cas-témoins sur une période de 6 mois allant de janvier à juin 2013. La population étudiée dans cette étude transversale était constituée d'enfants âgés de 6 à 59 mois hospitalisés pour paludisme grave confirmé. A été défini comme paludisme grave tout cas présentant au moins un critère de gravité de l'Organisation mondiale de la santé (OMS) [10] associé à une parasitémie périphérique positive à Plasmodium falciparum quelle que soit la densité parasitaire. Les enfants ayant constitué notre échantillon (*groupe I*) étaient hospitalisés aux urgences des cliniques universitaires de Kinshasa, au centre pédiatrique de Kalembe-Lembe dans la commune de Lingwala, au centre Hospitalier Lisungi dans la commune de Mont-Ngafula et à l'Hôpital militaire de référence du camp Tshatshi dans la commune de Ngaliema. Ils étaient âgés de 6 à 59 mois sans distinction de sexe. Nous avons pu réunir un total de 46 enfants (malades). Chaque cas a été apparié à un témoin avec un ratio égal à 1 sur l’âge, le sexe, la date d'hospitalisation et la commune de résidence. Les enfants ayant constitué le groupe témoin (*groupe II*) ont été choisis parmi des enfants supposés sains (avec une goutte épaisse négative) après avis favorables de leurs parents ou responsables.

Les prélèvements ont été effectués à jeun pendant la matinée de 8h00’ à 10h30’ dans les hôpitaux respectifs et les échantillons ont été analysés au laboratoire de Biochimie et Hématologie de la Faculté des Sciences Pharmaceutiques de l'Université de Kinshasa. Les données ont été colligées à partir des dossiers des patients hospitalisés. Une fiche d'enquête a été remplie pour tous les cas et témoins par le médecin chargé de l'enquête. Les variables suivantes ont été étudiées: taux sérique de la GOT, de la GPT, des bilirubines (totale, directe et indirecte) ainsi que l'hémoglobine. Ces variables ont été regroupés selon les valeurs normales établies [11, 12]. Les résultats obtenus ont été saisis et analysés sur le logiciel Epi Info 2011 (version 7.0.8.1). Les moyennes sont présentées avec les écarts-types et les Odds ratio (OR) avec un intervalle de confiance à 95% (IC 95%). Le test de Student a été utilisé pour la comparaison des moyennes et celle des fréquences (exprimées en pourcentage) par le test de X2 corrigé de Yates ou le test exact de Fisher lorsque recommandés. Le seuil de signification a été fixé à p <0,05. Considérations éthiques: La recherche pour réaliser ce travail a été autorisée par le comité d’éthique de l'Université de Lubumbashi. Un consentement libre et éclairé (verbal ou écrit) de toutes les personnes impliquées dans cette étude a été obtenu au préalable.

## Résultats

Le Tableau 1 nous montre la répartition des résultats d'examens sériques de nos patients comparés à ceux de témoins. La moyenne du taux de l'hémoglobine est de 6,7±2,6 g/dl allant de 2,8 à 12,7 g/dl dans le groupe I alors qu'elle est de 11,3±1,0 g/dl variant de 9,3 à 13,3 g/dl. Le test de Student donne une différence statistiquement significative en comparant ces deux moyennes (t = 11,21; p = 0,0000). En répartissant les résultats d'hémoglobine de notre échantillon en fonction des valeurs normales, il s'avère que près de 94% des enfants du groupe I présentent une anémie contre 30% chez les enfants du groupe II. La comparaison de ces deux proportions donne une différence statistique significative (p = 0,0000). S'agissant de l'activité GOT, la moyenne du taux des transaminases GOT est de 19,9±6,8 UI allant de 8 à 33,6 UI dans le groupe I alors qu'elle est de 5,4±3,3 UI variant entre 0,3 et 15,6 UI dans le second groupe. L'analyse statistique montre une différence significative en comparent ces deux moyennes(t = 12,95; p = 0,0000). En répartissant les résultats de la GOT en fonction des valeurs normales, il ressort que 41,3% des enfants du groupe I ont un taux quasiment élevé de la GOT contre aucun chez les enfants du groupe II. La comparaison de ces deux proportions donne une différence statistique significative (p = 0,0000).

**Tableau 1:** Comparaison des valeurs du bilan hépatique des enfants paludéens par rapport aux témoins

Paramètre	Groupe I (n = 46)		Groupe II (n = 46)		p	OR (IC95%)
	n	%	n	%		
**Hémoglobine (en g/dL)**						
<11,0	43	93,5	14	30,4	0,0000	32,8 (8,7-123,7)
≥11,0	3	6,5	32	69,6	-	1
Moyenne	6,7±2,6		11,3±0,9		0,0000	
**GOT (en UI/L)**						
<22	27	58,7	46	100	-	1
≥22	19	41,3	0	0,0	0,0000	Undefined
Moyenne	19,9±6,8		5,4±3,3		0,0000	
**GPT (en UI/L)**						
<22	10	21,7	44	95,6	-	1
≥22	36	78,3	2	4,4	0,0000	79,2 (16,3-384,8)
Moyenne	29,5±8,5		7,4±4,4		0,0000	
**Bilirubine totale (en mg/dL)**						
≤1,2	6	13,0	31	67,4	-	1
>1,2	40	87,0	15	32,6	0,0000	13,8 (4,8-39,6)
Moyenne	2,7±1,2		1,4±1,3		0,0000	
**Bilirubine directe (en mg/dL)**						
≤0,25	23	50,0	43	93,5	-	1
>0,25	23	50,0	3	6,5	0,0000	14,3 (3,9-52,9)
Moyenne	0,7±0,6		0,2±0,4		0,0000	
Bilirubine indirecte (en mg/dL)						
≤1,0	10	21,7	31	67,4	-	1
>1,0	36	78,3	15	32,6	0,00002	7,4 (2,9-18,9)
Moyenne	2,1±1,2		1,20±1,2		0,0003	

GOT: Glutamate Oxaloacétate Transaminase; GPT: Glutamate Pyruvate Transaminase

Concernant l'activité GPT, le taux moyen de la GPT est de 29,4±8,4 UI variant entre 10,3 et 42,3 UI dans le groupe I alors qu'il est de 7,4±4,3UI variant entre 1,31 et 23,6 UI dans le groupe II. Le test de Student montre une différence statistiquement significative en comparent les deux moyennes(t = 15,55; p= 0,0000). En fonction des valeurs de référence, il ressort que 78,3% des enfants du groupe I ont un taux élevé de la GPT contre 4,4% chez les enfants du groupe II et l'analyse statistique donne différence significative quand on compare ces proportions (p = 0,0000). En ce qui concerne la bilirubine totale, la moyenne du taux de la bilirubine totale est de 2,7±1,1 mg/dl allant de 0,9 à 5,5 mg/dl dans le groupe I alors qu'elle est de 1,4±1,3 mg/dl variant de 0,1 à 4,8 mg/dl dans le groupe II. Le test de Student donne une différence statistiquement significative en comparant ces deux moyennes (t = 5,25; p = 0,0000). Il s'avère que près de 87% des enfants du groupe I ont un taux élevé de la bilirubine totale contre 32,6% chez les enfants du groupe II. La comparaison de ces deux proportions donne une différence statistique significative (p = 0,0000). Le taux moyen de la bilirubine directe est de 0,7±0,7 mg/dl allant de 0,07 à 2,7 mg/dl dans le groupe I alors qu'il est de 0,2±0,4 mg/dl variant entre 0,01 et 2,8 mg/dl dans le groupe II. La différence entre ces deux moyennes set statistiquement significative (t = 4,31; p = 0,0000). Cinquante pourcent des enfants du groupe I ont un taux élevé de la bilirubine directe contre 6,5% chez les enfants du groupe II. La comparaison de ces deux proportions donne une différence statistique significative (p = 0,0000).

Enfin, le taux moyen de la bilirubine indirecte est de 2,1±1,2 mg/dl allant de 0,8 à 6,7 mg/dl dans le groupe I alors qu'il est de 1,2±1,1 mg/dl variant de 0,09 à 4,6 mg/dl dans le groupe II. La comparaison de ces deux moyennes donne une différence statistiquement significative (t = 3,76; p = 0,0003). Septante huit virgule trois pourcent des enfants du groupe I ont un taux élevé de la bilirubine indirecte contre 32,6% chez les enfants du groupe II et en comparant ces deux proportions, l'analyse donne une différence statistique significative (p = 0,00002).

## Discussion

Notre étude a révélé une diminution considérable de l'hémoglobine, au-delà de la valeur normale chez les enfants paludéens de moins de 5 ans; ceci pourrait être dû à un état d'hyper-hémolyse qui survient pendant la phase de schizogonie érythrocytaire chez ces derniers [9, 13]. Nous avons constaté également une élévation significative de l'activité des transaminases (GOT et GPT), ces valeurs sont au delà des limites physiologiques préconisées qui doivent être inferieures ou égales à 22 U/L. Ces données corroborent les observations faites au cours des études réalisées antérieurement par Shah et Iwalokun qui indiquent que dans tous les cas de paludisme graves; il y a une importante élévation des enzymes hépatiques. Une élévation modérée de la GOT et de la GPT peut refléter une discrète cytolyse due au cycle exo-érythrocytaire entretenu dans le foie par le plasmodium [14, 15]. Une étude réalisée par Patwari rapporte des taux élevés des enzymes aspartates transaminases et alanines transférases ont été constatées dans le sérum des enfants paludéens de moins de 3 ans, signifiant ainsi un dysfonctionnement hépatique [16]. Nous pensons que l'accroissement significatif des taux des transaminases est en relation avec l'accroissement de la parasitémie et cette relation est en corrélation avec le temps après infestation. Donc l’élévation de ces enzymes indique non seulement qu'il y a hémolyse mais aussi des lésions hépatiques [9].

Nos résultats ont également révélé une élévation significative des taux de bilirubine totale, bilirubine directe et indirecte chez les enfants atteints du paludisme grave. Nous pensons que la mesure de ces paramètres biologiques est justifiée dans le cas d'un paludisme grave car comme l'indiquent Dey, dans le cadre d'une évaluation de la fonction hépatique, la mesure des bilirubines directe et totale constituent des meilleurs indicateurs des lésions hépatiques après hémolyses. L'accroissement significatif de la bilirubine conjuguée dans le sérum infesté, est vraisemblablement consécutif à une cholestase intra-hépatique tandis que l'hyperbilirubinémie non conjuguée résulte de l'hémolyse intravasculaire des érythrocytes parasités [9]. Toutefois, nos résultats rejoignent ceux de Shah et Mohanty qui qui indiquent qu'une hyperbilirubinémie significative est constatée chez les enfants paludéens, traduisant une atteinte hépatique. Nous pensons ainsi que la bilirubine et les aminotransférases sont modérément élevées dans beaucoup de complications de la malaria à *Plasmodium falciparum* [14, 17]. L'hyperbilirubinémie est à l'origine de l'ictère chez l'enfant paludéen. Or, la présence d'un ictère est un des critères du paludisme grave de l'OMS car elle est significativement associée à une atteinte cérébrale, une insuffisance rénale aiguë et une importante parasitémie [11].

Signalons enfin que la sévérité de l'atteinte hépatique au cours du paludisme grave peut entraver le traitement même de ce paludisme à cause de la possible perturbation de la pharmacocinétique des antimalariques. En effet, l'effondrement de la clairance hépatique peut être à l'origine des concentrations sanguines élevées d'antimalariques avec un risque réel de surdosage. [12]. De la même manière, une diminution de la clairance hépatique des lactates peut être à l'origine de l’élévation de la lactémie, responsable d'une acidose lactique rencontrée plus fréquemment chez l'enfant impaludé. Par ailleurs, une insuffisance hépatocellulaire peut être à l'origine de l'hypoglycémie rencontrée dans le paludisme grave de l'enfant. Elle résulte souvent d'un défaut de la néoglucogenèse hépatique expliquée en partie par l'ischémie hépatique qui survient lors du paludisme grave chez l'enfant.

## Conclusion

Dans la présente étude, nous avons évalué l'atteinte de la fonction hépatique au cours du paludisme grave chez les enfants de moins de 5 ans et nous concluons à la lumière des résultats de notre étude que le paludisme grave à P. falciparum peut être responsable de l'atteinte hépatique. Cette atteinte résulte vraisemblablement de l'existence d'une phase pré-érythrocytaire hépatique obligatoire lors du cycle parasitaire de P. falciparum. A notre connaissance, il n'existe pas de traitement curatif spécifique de la défaillance hépatique. Néanmoins, deux conséquences possibles de la dysfonction hépatique peuvent être rapidement corrigées. Il s'agit de l'hypoglycémie et de l'acidose lactique pour lesquelles la correction fait utilement appel à l'exsanguino-transfusion. Le traitement même du paludisme grave chez l'enfant par antipaludéens devrait être couplé à un TDM (therapeutic drug monitoring) afin d’éviter de risques de surdosage résultant de la diminution de la clairance hépatique qui survient souvent au cours du paludisme grave chez l'enfant.
